# Psychological Aspects of Cutaneous Pain in Psoriasis

**DOI:** 10.3390/jcm13164890

**Published:** 2024-08-19

**Authors:** Magdalena Kotewicz, Piotr K. Krajewski, Andrzej K. Jaworek, Jacek C. Szepietowski

**Affiliations:** 1 University Centre of General Dermatology and Oncodermatology, Wroclaw Medical University, 50-368 Wroclaw, Poland; mazgajmagda@gmail.com (M.K.); piotr.krajewski@umw.edu.pl (P.K.K.); 2 Department of Dermatology, Jagiellonian University, 31-008 Kraków, Poland; andrzej.jaworek@uj.edu.pl

**Keywords:** psoriasis, cutaneous pain, depression

## Abstract

**Introduction:** Psoriasis is a chronic inflammatory disease that negatively impacts patients’ quality of life (QoL) and mental health. Itch and pain are prevalent symptoms of psoriasis and contribute to the psychosocial burden of this disease. This study aimed to evaluate the impact of skin pain on the prevalence and severity of symptoms of anxiety and depression and on the QoL in psoriasis patients. **Methods:** The studied population comprised 106 adults with psoriasis (34% female; mean age 42.1 ± 13.0 years). Disease severity was measured with the Psoriasis Area and Severity Index (PASI). The intensity of skin pain was assessed with the NRS and the Short Form McGill Pain Questionnaire (SF-MPQ). The Generalized Anxiety Disorder-7 (GAD-7) and Patient Health Questionnaire-9 (PHQ-9) questionnaires were used to estimate the severity of depression and anxiety, respectively, as was the Hospital Anxiety and Depression Scale (HADS). Quality of life (QoL) was studied using the Dermatology Life Quality Index (DLQI). **Results:** Regarding anxiety assessment, females reported significantly higher scores with the HADS-A (8.42 ± 4.85 points vs. 5.14 ± 3.9 points; *p* < 0.001) and the GAD-7 compared to men (7.50 ± 5.58 points vs. 5.24 ± 4.79 points; *p* = 0.036). Similarly, the severity of depression was significantly higher in women, as measured with the PHQ-9 (7.50 ± 5.58 points vs. 5.24 ± 4.79 points, *p* = 0.021). Psoriasis patients with skin pain scored significantly higher in HADS Total score (*p* = 0.043), HADS-A (*p* = 0.022), PHQ-9 (*p* = 0.035), and DLQI (*p* < 0.001) than the rest of the studied group. The intensity of skin pain measured with the SF-MPQ correlated significantly with HADS Total score (*p* = 0.021), HADS-A (*p* < 0.001), HADS-D (*p* = 0.038), and PHQ-9 (*p* < 0.001). Additionally, there was a significant correlation between the intensity of cutaneous pain assessed using the VAS and the PHQ-9 (*p* = 0.022). **Conclusions:** Skin pain significantly influences the well-being of patients with psoriasis as well as the symptoms of anxiety and depression. In particular, women with psoriasis are at increased risk of developing anxiety and depression. Our findings underline the necessity for a multidisciplinary approach to the management of this dermatosis.

## 1. Introduction

Pain is defined as “an unpleasant sensory or emotional experience associated with or resembling that associated with actual or potential tissue damage”. Pain can be categorized as acute, which serves an adaptive survival function, or chronic, which is considered a pathological condition [1]. Chronic pain is frequently accompanied by psychiatric comorbidities, including anxiety and depression, insomnia, somatic symptom disorder, substance use disorders, and personality disorders [2,3]. Skin pain is a very prevalent symptom in many dermatological diseases, including hidradenitis suppurativa (83.6%), leg ulcers (67.6%), atopic dermatitis (54.7%), hand eczema (28.3%), rosacea (19.3%), psoriasis (19.1%), eczema (18.1%), and acne (15.3%) [4,5,6].

Psoriasis is a frequent chronic inflammatory disorder affecting approximately 2–3% of the global population [7]. It is characterized by the presence of erythematous, scaly patches and plaques located on the trunk, extremities, scalp, and face. Compared to other chronic diseases, only depression and chronic lung disease exert a more negative impact on quality of life than psoriasis [8]. Health-related quality of life is often defined as a subjective perception of the value of life influenced by diseases, disabilities, treatments, and functional states [9]. Due to the visible nature of the lesions, psoriasis patients often feel stigmatized and may avoid intimacy [10]. In particular, palmoplantar involvement results in more significant disability and discomfort in individuals with psoriasis [11]. In addition, psoriatic arthritis exerts a negative impact on sleep, work, and socialization [12]. Therefore, psychological comorbidities are common in patients with this dermatosis [13]. The symptoms, including itch, irritation, burning, sensitivity, and pain, are frequently reported in psoriasis [14]. Itch is recognized as one of the most burdensome symptoms of this disease that impairs health-related quality of life and can lead to stigmatization, anxiety, and depression [15]. Nevertheless, the effects of cutaneous pain on mental health in individuals with psoriasis remain largely unexplored. Thus, the objective of the current study was to assess the impact of skin pain on the prevalence of depression, anxiety, quality of life, and stigmatization in psoriasis patients.

## 2. Materials and Methods

This study was conducted with a cohort of 106 patients recruited from private practices; the Department of Dermatology, Venerology, and Allergology of Wroclaw Medical University in Wroclaw, Poland; and the Department of Dermatology at the University Hospital in Cracow, Poland, between March 2023 and January 2024. A dermatologist examined all patients, and demographic data were collected. Patients were included if they had psoriasis, had not received systemic treatment for psoriasis for at least 3 months, had not used topical treatment for at least 1 month except for emollients, and could read and write Polish. The exclusion criteria included age younger than 18 years, another concomitant dermatological disease associated with pain, psoriatic arthritis, a psychiatric diagnosis, a cognitive impairment that affects the ability to complete self-report questionnaires, and an uncertain diagnosis. This study was performed in accordance with guidelines for human studies and the World Medical Association of Helsinki.

### 2.1. Disease Severity Assessment

The Psoriasis Area and Severity Index (PASI) was used to assess the severity of the disease, with a scoring range of 0 to 72 points. The PASI evaluates lesions by considering factors such as erythema, scaling, induration, and the affected surface area [16].

### 2.2. Pain Assessment

A numerical rating scale (NRS) was used in order to assess skin pain intensity. Patients with psoriasis were asked to evaluate the severity of the most intense skin pain experienced in the past week. The NRS was a one-dimensional tool for measuring symptom intensity ranging from 0 (no pain) to 10 (the worst imaginable pain) [17]. The established cut-off points for the NRS were as follows: ≤5 points indicate mild pain, >5 to 7 points represent moderate pain, and >7 to 10 points account for severe pain [18].

Another instrument used to assess skin pain intensity was the Short Form McGill Pain Questionnaire (SF-MPQ) [19]. This tool consists of 15 descriptors, including 11 sensory descriptors and 4 affective descriptors. The Pain Rating Index comprises two subscales: an 11-item sensory subscale and a 4-item affective subscale, with each item rated on a scale from 0 (none) to 3 points (severe) [16,17]. Finally, a horizontal visual analogue scale (VAS) was utilized for measuring average pain, which ranges from 0, denoting “no pain”, to 10 points, representing “worst imaginable pain” [20].

### 2.3. Psychosocial Burden

To assess the psychosocial burden of skin pain in psoriasis, all participants were required to fulfill a questionnaire including the Hospital Anxiety and Depression Scale (HADS), the Generalized Anxiety Disorder-7 (GAD-7), the Patient Health Questionnaire-9 (PHQ-9), the Dermatology Life Quality Index (DLQI), and the 6-Item Stigmatization Scale (6-ISS).

#### 2.3.1. Hospital Anxiety and Depression Scale

The HADS is a 14-item self-administered questionnaire designed to assess the symptoms of anxiety (HADS Anxiety, HADS-A) and depression (HADS Depression, HADS-D) during the past week. It includes seven queries related to depression and seven queries related to anxiety, each scoring from 0 to 3 points. The maximum total score is 42 points, with each subscale having a maximum score of 21 points. A score of 11 points or higher on the overall scale indicates an abnormal result, while scores of 8 points or greater on each subscale suggest a diagnosis of depression or anxiety [21,22].

#### 2.3.2. Generalized Anxiety Disorder-7

The GAD-7 is a widely utilized screening instrument for generalized anxiety disorder. The questionnaire consists of 7 questions that assess anxiety symptoms experienced over the past two weeks. Each question is scored on a 4-point scale, with 0 points for “not at all”, 1 point for “several days”, 2 points for “over half the days”, and 3 points for “nearly every day”. The total score is calculated by adding up the points from each of the seven questions. Score cut-offs of 5, 10, and 15 points indicate mild, moderate, and severe anxiety levels, respectively [23].

#### 2.3.3. Patient Health Questionnaire-9

The PHQ-9 is a 9-item instrument developed for the screening of depression in various healthcare settings. Patients evaluate the frequency of each of the nine diagnostic criteria for major depressive disorder, as outlined in the Diagnostic and Statistical Manual of Mental Disorders, Fourth Edition (DSM-IV). The response options are scored from 0, “not at all”, to 3 points, “nearly every day”. Standard cut-off scores of 5, 10, 15, and 20 points correspond to mild, moderate, moderately severe, and severe depression, respectively. In the context of depression screening, a score of 10 points or greater was defined as the cut-off for the possibility of major depressive disorder [24].

#### 2.3.4. Dermatology Life Quality Index

The patient’s quality of life (QoL) was evaluated using a Polish version of the Dermatology Life Quality Index (DLQI) questionnaire. The DLQI is a specialized tool designed for dermatological conditions. It assesses various aspects over the past week, including daily activities, symptoms and feelings, leisure, personal relationships, work- and school-related matters, and treatment adverse effects. The questionnaire consists of 10 items, with each item scored on a scale from 0 to 3 points (0 meaning “not at all”, 1 point meaning “a little”, 2 points meaning “a lot”, and 3 points meaning “very much”). The individual scores are then summed up to calculate the total DLQI score, which spans from 0 to 30 points. The interpretation of the total DLQI score is as follows: 0–1 point represents a minimal impact on QoL, 2–5 points suggest a small impact, 6–10 points indicate a moderate impact, 11–20 points signify a large impact, and 21–30 points denote an extremely large impact on the patient’s quality of life [22].

#### 2.3.5. 6-Item Stigmatization Scale

The 6-ISS is a dermatology-specific instrument used to assess perceived stigmatization owing to skin disease. It consists of 6 questions that the patient answers on a 4-point scale: 0 points meaning “not at all”, 1 point indicating “sometimes”, 2 points representing “very often”, and 3 points meaning “always”. The six dimensions of stigmatization include anticipation of rejection, sensitivity to the opinions of others, feelings of being flawed, negative attitudes, guilt and shame, and secretiveness. The total score ranges from 0 to 18 points, with higher scores indicating a greater degree of perceived stigmatization experienced by the patient due to skin conditions [24,25,26].

### 2.4. Statistical Analysis

Before conducting the analysis, all data were checked for normality using the Shapiro–Wilk test. For each variable, the minimum, maximum, mean, and standard deviation were calculated. Based on the normality of the data distribution, quantitative variables between two groups were compared using either the T-Student test or the Mann–Whitney U test. Correlations were evaluated using Spearman and Pearson coefficients. The Chi-squared test was used for qualitative data. Differences among more than two groups were assessed using either ANOVA or the Kruskal–Wallis test. Statistical significance was determined with a two-sided *p* value of ≤0.05. All statistical analyses were performed using IBM SPSS Statistics version 26 (SPSS Inc., Chicago, IL, USA).

## 3. Results

The study group comprised 106 patients with psoriasis, consisting of 36 females (34.0%) and 70 males (66.0%), with an age range of 18–72 (average 42.07 ± 12.96) years. The mean PASI score was 10.93 ± 8.47 points, while the average disease duration was 14.89 years (SD ± 12.69), ranging from 1 to 55 years. Among all 106 subjects, 53 (50%) reported cutaneous pain in the past week, involving 55.6% of females (*n* = 20) and 47.1% of males (*n* = 33), with a mean worst pain intensity of 2.42 ± 2.96 points on the NRS. In the studied population, the intensity of skin pain measured with the SF-MPQ was 4.84 ± 7.51 points, with the sensory component being equal to 3.85 ± 6.03 points and the affective component being 0.99 ± 1.89 points, without any significant difference between the genders. The average VAS score was 1.92 ± 2.65 points in the whole cohort. The data are presented in Table 1.

The mean HADS Total score for the whole studied population was 10.40 ± 7.47 points, with significantly higher (*p* = 0.005) scores in women (13.11 ± 7.93 points) than in men (9.00 ± 6.86 points).

Regarding anxiety assessment, the mean HADS-A score was 6.25 ± 4.52 points, while the average GAD-7 value was 6.01 ± 5.16 points in the whole group. A statistically significant difference between females and males was found in the HADS-A score (8.42 ± 4.85 points vs. 5.14 ± 3.9 points; *p* < 0.001) and the GAD-7 score (7.50 ± 5.58 points vs. 5.24 ± 4.79 points; *p* = 0.036). Regarding depression assessment, the mean HADS-D score was 4.14 ± 3.68 points, and the average PHQ-9 score was 6.86 ± 5.98 points in the whole group. There was a statistically significant difference in the PHQ-9 scores between women and men (7.50 ± 5.58 points vs. 5.24 ± 4.79 points, *p* = 0.021), but no such difference was observed in the HADS-D scores (4.69 ± 3.85 points vs. 3.86 ± 3.58 points; NS). As far as the assessment of quality of life and stigmatization is concerned, the mean DLQI score was 8.09 ± 6.99 points and the average 6-ISS score was 4.72 ± 3.63 points, but no statistically significant differences between females and males were noted regarding these parameters (Table 2).

Psoriasis patients who reported skin pain in the past week achieved significantly higher scores with the HADS Total score (*p* = 0.043) and the HADS-A (*p* = 0.022) than the rest of the studied population. Additionally, anxiety assessed with the HADS-A was diagnosed in 22 patients with skin pain (41.5%), and it was significantly more prevalent than in patients without skin pain (22.6%) (*p* = 0.037). However, there was no statistically significant difference in the mean GAD-7 values between both studied groups and there was no significant difference in the distribution of anxiety disorder severity groups (as measured with the GAD-7) between patients with and without pain (Table 3 and Table 4).

**Figure 1:**
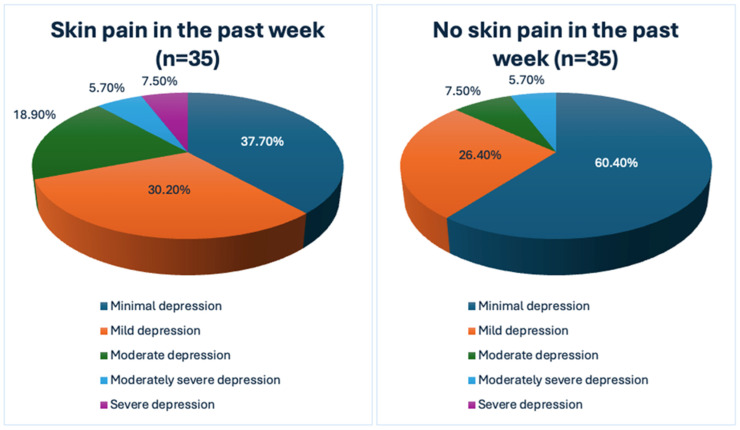
The impact of skin pain on the severity of depression as evaluated by the Patient Health Questionnaire-9 (PHQ-9).

Regarding depression assessment, psoriatic individuals with cutaneous pain scored significantly higher with the PHQ-9 than those without pain (*p* = 0.035) and the distribution of depression severity groups (according to PHQ-9 scores) was statistically different between both studied groups (*p* = 0.05) (Figure 1). However, there was no significant difference in the mean HADS-D scores between patients with and without pain.

**Figure 2:**
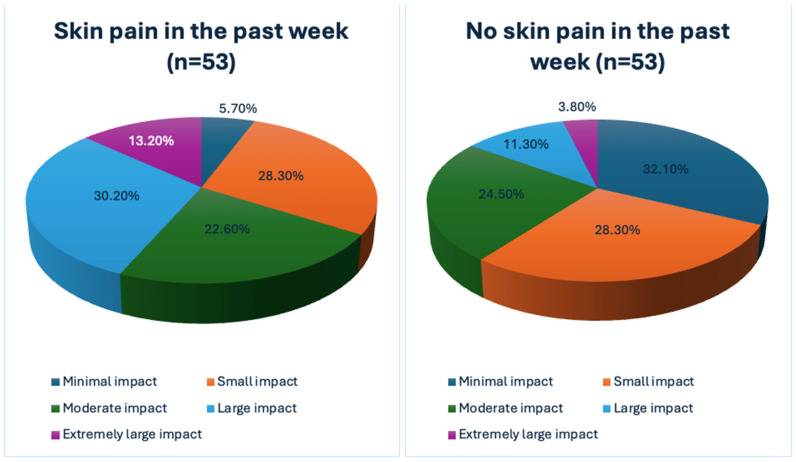
The impact of skin pain on the quality of life.

Additionally, psoriasis patients with skin pain scored significantly higher with the DLQI than participants without skin pain (*p* < 0.001) and there was a statistically significant difference in the distribution of quality of life scores between the studied groups (*p* = 0.002) (Figure 2). There was no statistically significant difference in assessing stigmatization using the 6-ISS between patients with skin pain and the rest of the participants (Table 3 and Table 4).

Although there were no correlations between the NRS pain assessment and the studied psychosocial parameters, the intensity of skin pain measured with the SF-MPQ Total score correlated significantly with HADS Total score (*p* = 0.021), HADS-A (*p* < 0.001), HADS-D (*p* = 0.038), and PHQ-9 (*p* < 0.001). Similarly, the sensory component of the SF-MPQ also correlated significantly with HADS Total score (*p* = 0.038), HADS-A (*p* = 0.001), HADS-D (*p* = 0.037), and PHQ-9 (*p* < 0.001). Correspondingly, the affective subscale of the SF-MPQ correlated significantly with HADS Total score (*p* = 0.039), HADS-A (*p* < 0.001), and PHQ-9 (*p* < 0.001). Additionally, there was a significant correlation between the intensity of cutaneous pain measured using the VAS elements of the SF-MPQ and the PHQ-9 (*p* = 0.022) (Table 5).

## 4. Discussion

Psychiatric disorders among psoriasis patients have already been analyzed in numerous publications. Evidence from systematic reviews suggests that psoriasis is associated with depression, anxiety, sleep disorders, personality disorders, substance-related and addictive disorders, schizophrenia, bipolar disorder, and suicidal ideations [27].

A meta-analysis involving 401,703 psoriasis patients demonstrated that individuals with this disease were at least one and a half times more prone to manifest depressive symptoms than their healthy peers [28]. According to the HADS questionnaires, the prevalence of symptoms of depression was 23%, using the cut-off value of eight [28]. In comparison, the prevalence of clinical depression was 12%, according to the International Classification of Disease codes [28]. Another recent systematic review demonstrated that females with psoriasis and subjects affected by psoriatic arthritis were more prone to depression than their male counterparts and those without psoriatic arthritis, respectively [29]. These findings support the results of our study, in which we reported more prevalent symptoms of depression in women with psoriasis compared to men. The authors of another systematic review noted that most observational studies reported a higher overall prevalence of depression among females with psoriasis than men with the same condition. However, when comparing patients of both sexes with psoriasis to control groups, the prevalence of depression was still found to be increased in men with psoriasis compared to their non-psoriatic counterparts. Therefore, they concluded that the frequency of depression is higher in individuals with psoriasis despite gender, though being a woman may be a supplementary risk factor [30].

The symptoms of anxiety in psoriasis seem to be even more prevalent than the symptoms of depression. In the meta-analysis by Jalenques I. et al., 34% of psoriasis patients demonstrated the symptoms of anxiety, according to questionnaires including the HADS, and the prevalence of anxiety symptoms decreased with age [31]. Depending on the type of anxiety disorder, the prevalence was 15% for social anxiety disorder, 11% for generalized anxiety disorder, and 9% for unspecified anxiety disorder [31]. There was a significant association between anxiety symptoms and psoriasis [31]. In another meta-analysis, the female sex was associated with increased anxiety in psoriasis patients compared to men with this disease, which is consistent with the results of our study. Additional risk factors for the development of anxiety in this systematic review included moderate and severe psoriasis and psoriatic arthritis [29].

Psoriasis and mental illnesses share immunological changes that lead to high proinflammatory cytokine levels, which is known as “the cytokine hypothesis” [32]. The pathogenesis of psoriasis involves complex interplays between components of the immune system, including dendritic cells and T-helper (Th) lymphocytes, and the skin, particularly keratinocytes. Dysregulation of various cytokine-signaling molecules—such as interferon-gamma (IFN-γ), tumor necrosis factor (TNF), and interleukin (IL)-6, IL-12, IL-17, IL-22, and IL-23—within the skin can drive inflammatory responses and the development of psoriatic lesions. Specifically, the psoriatic skin exhibits elevated levels of IL-6, which can promote the differentiation of proinflammatory Th17 cells while concurrently inhibiting regulatory T (Treg) cells. This disruption of the Th17/Treg balance consequently changes the normal differentiation of keratinocytes, further contributing to the maintenance of psoriasis [33].

The inflammation in psoriasis, however, is not limited exclusively to the skin, as elevated cytokine levels are found in the blood of the affected individuals [32]. A meta-analysis found that levels of IL-6 and TNF are increased in the blood of depression patients [34]. In addition, there is an association between the serum levels IL-23 and IL-17 and the severity of the depression or anxiety symptoms [35,36]. Moreover, antidepressant treatment decreases the serum levels of TNF-α [37]. Additionally, the successful treatment of psoriasis with biologics such as tumor necrosis factor inhibitors or interleukin 12/23 inhibitors is associated with a significant reduction in depression symptoms [38]. Hyperactivation of the hypothalamus–pituitary–adrenal (HPA) axis is another mechanism by which cytokines may lead to depression. Studies on transgenic mice demonstrated that IL-6 is overproduced in the central nervous system (CNS) in response to stress [39], suggesting that elevated IL-6 in the CNS can predispose individuals to hyperactivation of the HPA axis [33].

In psoriasis patients, the frequency of skin pain varies between 19.1% and 51.9%, which is in line with the results of our study, and is most often of moderate intensity [39,40,41,42,43,44]. Cutaneous pain in psoriasis can be elucidated by the process of neurogenic inflammation, where mediators released from peripheral sensory neurons directly recruit and activate adaptive immune cells (T lymphocytes) and innate immune cells, such as mast cells and dendritic cells [45,46]. As a result, mast cells release mediators involving cytokines such as IL-5, IL-6, IL-1ß, tumor necrosis factor (TNF), serotonin, tryptase, histamine, and nerve growth factor (NGF), which commence the mutual communication with nociceptors located on sensory nerve fibers. These nerve fibers then discharge inflammatory and vasoactive neuropeptides, activating mast cells through a feedback mechanism [47].

There are limited data on the influence of skin pain on psychometric parameters in psoriasis. In the survey by Misery et al. [41], 33.2% of participants reported skin pain in a studied group of 244 psoriasis patients. It was demonstrated that individuals with painful psoriasis had significantly higher scores using the mental composite score-12 (39.18 vs. 42.97, respectively; *p* < 0.05) and the DLQI than healthy controls (13.62 vs. 7.66, respectively; *p* < 0.000001).

In the study by Ljosaa et al. [42], conducted on 139 psoriasis patients, 41.7% of participants reported skin pain in the past 24 h, while 36.7% perceived skin discomfort. Skin pain was associated significantly with health-related quality of life (HRQoL). The mean DLQI score in the group of psoriasis patients with skin pain was 13.7 points (±4.4 points), which is higher than in our study. Additionally, the authors concluded that sleep disturbance served as a partial mediator in the relationship between skin pain and HRQoL.

In the study by Garcia-Fernandez et al. [43] on 119 psoriasis patients, 37.8% of participants reported skin pain, with a mean intensity of 5.7 ± 2.6 points on the VAS score. The mean DLQI score was 14.4 ± 6.9 points. DLQI scores were significantly higher in patients with skin pain, which is consistent with the results of our study, and correlated significantly with the VAS score. No association existed between the prevalence or intensity of skin pain and the PASI, static the Physician Global Assessment, or the Body Surface Area.

The impact of skin pain on mental health was investigated in other dermatoses. For instance, Vakharia et al. [48] conducted research on 144 patients with atopic dermatitis (AD), in which 42.7% of participants reported skin pain in the past week, involving 42 (13.8%) with severe or very severe pain. Skin pain severity was more strongly correlated with the DLQI (r = 0.45) and the Patient Health Questionnaire-9 (r = 0.36) (*p* < 0.0001 for both) than in our research. Additionally, strong correlations between skin pain and the Patient Oriented Eczema Measure (r = 0.54), the Itchy Quality of Life (r = 0.52), the 5-dimensions of itch scale (r = 0.47), the NRS for itch (r = 0.43) and sleep (r = 0.36), the patient-reported global AD severity (r = 0.34), the Eczema Area and Severity Index (r = 0.23), and the objective Scoring AD index (r = 0.20) (*p* < 0.0001 for all) were identified. In particular, skin pain exerted a negative influence on the capacity to shop and execute other activities of everyday life, make clothing choices, engage in social interactions or leisure activities, study or work, participate in sports, maintain relationships with others, experience normal sexual function, and manage treatment-related burdens (*p* ≤ 0.017 for all).

Regarding hand eczema (HE), Zalewski et al. [49] performed a study on a group of 100 patients with this disease. The average DLQI score in the cohort was 11.62 ± 6.35 points, higher than in our study. Similar to our findings, the severity of depressive symptoms, assessed by the PHQ-9 and HADS-M depression (D) questionnaires, correlated significantly with the intensity of skin pain (r = 0.445, *p* < 0.001 and *r* = 0.287, *p* =0.004, respectively). In addition, there were positive correlations between the severity of anxiety symptoms, as measured by the GAD-7 and HADS-M anxiety (A) instruments, and the intensity of skin pain (*r* = 0.248, *p* = 0.013 and *r* = 0.342, *p* = 0.001, respectively).

Regarding the limitations of our study, although we used only validated psychometric instruments, we observed some discrepancies in the assessments of anxiety and depression using different questionnaires. The possible explanation is that some tools differ in the content and the length of the studied period. For instance, four out of nine items in the PHQ-9 address somatic symptoms, while none of the seven HADS-D items investigate somatic symptoms. Additionally, the PHQ-9 and the GAD-7 examine the period of the past two weeks, whereas the HADS involves a time frame of one week [50]. Increasing the group size would clarify these differences. Furthermore, a detailed psychiatric examination will add value to confirm the existence of symptoms of psychiatric disorders. The strength of our study is that we used numerous pain and psychometric assessment tools to evaluate psoriatic patients, which, to our knowledge, has not been done till now.

In conclusion, our findings demonstrate the significant influence of skin pain on the well-being of patients with psoriasis, as well as the severity of anxiety and depression in this population. Our findings underline the importance of screening for the symptoms of anxiety and depression, particularly in women with psoriasis, and the necessity for a multidisciplinary approach in the management of this disease. Understanding the interplay between disease symptoms and psychological factors can enhance the early detection of psychiatric disorders, facilitating timely prevention of mental health issues and their repercussions. Moreover, effective management of these comorbid conditions may lead to improvements in psoriasis. A deeper awareness of both the physical and psychological burdens of the disease can help to customize treatment options more effectively and enhance patient compliance.

## Figures and Tables

**Table 1:** Group characteristics.

Characteristics	Whole Population (*n* = 106)	Females (*n* = 36)	Males (*n* = 70)	*p*
Age, years (mean ± SD)	42.07 ± 12.96	39.94 ± 14.00	43.16 ± 12.35	NS
PASI [points] (mean ± SD)	10.93 ± 8.47	10.18 ± 9.87	11.32 ± 7.71	NS
Disease duration, years (mean ± SD)	14.89 ± 12.69	15.00 ± 12.23	14.83 ± 13.00	NS
Family history of psoriasis	50 (47.2%)	21 (58.3%)	29 (41.4%)	NS
Pain in the past week	53 (50%)	20 (55.6%)	33 (47.1%)	NS
Pain NRS in the past week [points] (mean ± SD)	2.42 ± 2.96	3.06 ± 3.30	2.09 ± 2.73	NS
SF-MPQ Total score [points] (mean ± SD)	4.84 ± 7.51	5.89 ± 8.05	4.30 ± 7.22	NS
SF-MPQ sensory [points] (mean ± SD)	3.85 ± 6.03	4.39 ± 6.17	3.57 ± 5.99	NS
SF-MPQ affective [points] (mean ± SD)	0.99 ± 1.89	1.50 ± 2.24	0.73 ± 1.63	NS
VAS [points] (mean ± SD)	1.92 ± 2.65	2.32 ± 2.93	1.72 ± 2.48	NS

PASI—Psoriasis Area and Severity Index; NRS—numerical rating scale; SD—standard deviation; SF-MPQ—Short Form McGill Pain Questionnaire; VAS—visual analogue scale; NS—not significant.

**Table 2:** Psychosocial burden of skin pain in the whole population, males and females.

Characteristic (Mean ± SD)	Whole Population (*n* = 106)	Females (*n* = 36)	Males (*n* = 70)	*p*
HADS Total score [points]	10.40 ± 7.47	13.11 ± 7.93	9.00 ± 6.86	0.005
HADS-A [points]	6.25 ± 4.52	8.42 ± 4.85	5.14 ± 3.92	<0.001
HADS-D [points]	4.14 ± 3.68	4.69 ± 3.85	3.86 ± 3.58	NS
GAD-7 [points]	6.01 ± 5.16	7.50 ± 5.58	5.24 ± 4.79	0.036
PHQ-9 [points]	6.86 ± 5.98	8.61 ± 6.18	5.96 ± 5.71	0.021
DLQI [points]	8.09 ± 6.99	9.83 ± 7.70	7.20 ± 6.47	NS
6-ISS [points]	4.72 ± 3.63	5.39 ± 4.14	4.37 ± 3.32	NS

SD—standard deviation; HADS—Hospital Anxiety and Depression Scale; A—anxiety; D—depression; GAD-7—Generalized Anxiety Disorder-7; PHQ-9—Patient Health Questionnaire-9; DLQI—Dermatology Life Quality Index; 6-ISS—6-Item Stigmatization Scale, NS—not significant.

**Table 3:** Differences between psoriasis patients with and without skin pain.

Characteristic (Mean ± SD)	Skin Pain in the Past Week (*n* = 53)	No Skin Pain in the Past Week (*n* = 53)	*p*
HADS Total score [points]	12.17 ± 8.47	8.62 ± 5.868	0.043
HADS-A [points]	7.42 ± 5.09	5.09 ± 3.54	0.022
HADS-D [points]	4.75 ± 4.13	3.53 ± 3.08	NS
GAD-7 [points]	6.62 ± 5.65	5.40 ± 4.59	NS
PHQ-9 [points]	8.21 ± 6.72	5.51 ± 4.83	0.035
DLQI [points]	10.58 ± 7.29	5.60 ± 5.73	<0.001
6-ISS [points]	5.23 ± 3.76	4.21 ± 3.50	NS

SD—standard deviation; HADS—Hospital Anxiety and Depression Scale; A—anxiety; D—depression; GAD-7—Generalized Anxiety Disorder-7; PHQ-9—Patient Health Questionnaire-9; DLQI—Dermatology Life Quality Index; 6-ISS—6-Item Stigmatization Scale; NS—not significant.

**Table 4:** Prevalence of anxiety, depression, and impact on the quality of life.

Characteristic (Mean ± SD)	Skin Pain in the Past Week (*n* = 53)	No Skin Pain in the Past Week (*n* = 53)	*p*
HADS abnormal score	20 (37.7%)	7 (13.2%)	0.004
Anxiety			
HADS-A	22 (41.5%)	12 (22.6%)	0.037
GAD-7	16 (30.2%)	22 (41.5%)	NS
Minimal anxiety	24 (45.3%)	24 (45.3%)	NS
Mild anxiety	15 (28.3%)	22 (41.5%)	
Moderate anxiety	9 (17.0%)	4 (7.5%)	
Severe anxiety	5 (9.4%)	3 (5.7%)	
Depression			
HADS-D	11 (20.8%)	5 (9.4%)	NS
PHQ-9	18 (34.0%)	12 (22.6%)	NS
Minimal depression	20 (37.7%)	32 (60.4%)	0.05
Mild depression	16 (30.2%)	14 (26.4%)	
Moderate depression	10 (18.9%)	4 (7.5%)	
Moderately severe depression	3 (5.7%)	3 (5.7%)	
Severe depression	4 (7.5%)	0 (0%)	
Quality of life			
Minimal impact	3 (5.7%)	17 (32.1%)	0.002
Small impact	15 (28.3%)	15 (28.3%)	
Moderate impact	12 (22.6%)	13 (24.5%)	
Large impact	16 (30.2%)	6 (11.3%)	
Extremely large impact	7 (13.2%)	2 (3.8%)	

SD—standard deviation; HADS—Hospital Anxiety and Depression Scale; A—anxiety; D—depression; GAD-7—Generalized Anxiety Disorder-7; PHQ-9—Patient Health Questionnaire-9; DLQI—Dermatology Life Quality Index; 6-ISS—6-Item Stigmatization Scale; NS—not significant.

**Table 5:** Correlations between skin pain intensity and psychometric assessments.

	Pain NRS in the Past Week	SF-MPQ Total Score	SF-MPQ Sensory	SF-MPQ Affective	VAS
HADS Total score	NS	*p* = 0.021	*p* = 0.038	*p* = 0.039	NS
HADS-A	NS	*p* < 0.001	*p* = 0.001	*p* < 0.001	NS
HADS-D	NS	*p* = 0.038	*p* = 0.037	NS	NS
GAD-7	NS	NS	NS	NS	NS
PHQ-9	NS	*p* < 0.001	*p* < 0.001	*p* < 0.001	*p* = 0.022

HADS—Hospital Anxiety and Depression Scale; A—anxiety; D—depression; GAD-7—Generalized Anxiety Disorder-7; PHQ-9—Patient Health Questionnaire-9; DLQI—Dermatology Life Quality Index; 6-ISS—6-Item Stigmatization Scale; NRS—numerical rating scale; SD—standard deviation; SF-MPQ—Short Form McGill Pain Questionnaire; VAS—visual analogue scale; NS—not significant.

## Data Availability

The datasets analyzed or generated in the current study are available from the corresponding author upon reasonable request.
